# Circulating Ouabain Modulates Expression of Claudins in Rat Intestine and Cerebral Blood Vessels

**DOI:** 10.3390/ijms21145067

**Published:** 2020-07-17

**Authors:** Alexander G. Markov, Arina A. Fedorova, Violetta V. Kravtsova, Anastasia E. Bikmurzina, Larisa S. Okorokova, Vladimir V. Matchkov, Valeria Cornelius, Salah Amasheh, Igor I. Krivoi

**Affiliations:** 1 Department of General Physiology, St. Petersburg State University, 199034 St. Petersburg, Russia; a.markov@spbu.ru (A.G.M.); rishagod7@gmail.com (A.A.F.); v.kravtcova@spbu.ru (V.V.K.); st058627@student.spbu.ru (A.E.B.); l.okorokova@spbu.ru (L.S.O.); 2 Department of Biomedicine, University of Aarhus, C 8000 Aarhus, Denmark; vvm@biomed.au.dk; 3 Institute of Veterinary Physiology, Freie Universität Berlin, 14163 Berlin, Germany; valeria.cornelius@fu-berlin.de (V.C.); salah.amasheh@fu-berlin.de (S.A.)

**Keywords:** Na,K-ATPase, circulating ouabain, claudins, intestine, brain blood vessels, IPEC-J2 cells

## Abstract

The ability of exogenous low ouabain concentrations to affect claudin expression and therefore epithelial barrier properties was demonstrated previously in cultured cell studies. We hypothesized that chronic elevation of circulating ouabain in vivo can affect the expression of claudins and tight junction permeability in different tissues. We tested this hypothesis in rats intraperitoneally injected with ouabain (1 μg/kg) for 4 days. Rat jejunum, colon and brain frontal lobes, which are variable in the expressed claudins and tight junction permeability, were examined. Moreover, the porcine jejunum cell line IPEC-J2 was studied. In IPEC-J2-cells, ouabain (10 nM, 19 days of incubation) stimulated epithelial barrier formation, increased transepithelial resistance and the level of cSrc-kinase activation by phosphorylation, accompanied with an increased expression of claudin-1, -5 and down-regulation of claudin-12; the expression of claudin-3, -4, -8 and tricellulin was not changed. In the jejunum, chronic ouabain increased the expression of claudin-1, -3 and -5 without an effect on claudin-2 and -4 expression. In the colon, only down-regulation of claudin-3 was observed. Chronic ouabain protected the intestine transepithelial resistance against functional injury induced by lipopolysaccharide treatment or by modeled acute microgravity; this regulation was most pronounced in the jejunum. Claudin-1 was also up-regulated in cerebral blood vessels. This was associated with reduction of claudin-3 expression while the expression of claudin-5 and occludin was not affected. Altogether, our results confirm that circulating ouabain can functionally and tissue-specifically affect barrier properties of epithelial and endothelial tissues via Na,K-ATPase-mediated modulation of claudins expression.

## 1. Introduction

Na,K-ATPase is a member of the P-type ATPase superfamily, a vital transport protein that ubiquitously expressed in the plasma membrane of all animal cells. The Na,K-ATPase is responsible for establishing Na^+^ and K^+^ ions transmembrane gradient that provides membrane excitability and the driving force for many Na^+^-dependent secondary transporters. The functional enzyme is a heteromer composed of α subunit, responsible for catalytic and transport function, and β subunit, which is required for enzymatic activity and modulates the enzyme affinity to Na^+^ and K^+^ ions. In some tissues a small protein of FXYD family has been found as an auxiliary subunit that modulates Na,K-ATPase activity. Four α isoforms, three β isoforms, and seven FXYD isoforms, encoded by separate genes and expressed in a cell- and tissue-specific manner have been identified that provide a wide molecular and functional diversity of the Na,K-ATPase. In mammals, the α1 isoform is ubiquitously expressed, while the α2–α4 isoforms show a more restricted cellular and subcellular distribution. The majority of cell types co-express the α1 isoform in combination with other α isoforms. However, in erythrocytes, kidney and intestine epithelia the sole α1 isoform is expressed [1,2,3,4,5].

The extracellular loops of α subunit form a unique highly specific binding site for cardiotonic steroids (CTS) that act as the Na,K-ATPase inhibitors. CTS found in plants and animal tissues and characterized by a steroid nucleus. Ouabain and marinobufagenin are the most known among them [2,6,7,8]. The Na,K-ATPase is also known as a scaffolding protein that is able to form multimolecular complexes located in specialized sub-cellular microdomains. This allows the Na,K-ATPase to perform a number of functions that are not directly related to ion distribution across the plasma membrane. Particularly, an intracellular domain of α subunit that mediates specific protein–protein interactions with cSrc-kinase and its signaling function was shown [9,10]. Currently, the α1Na, K-ATPase/cSrc complex is recognized as a receptor for CTS that initiates downstream a variety of intracellular signaling pathways. Thus, the Na,K-ATPase is now considered as an important signaling molecule in neuronal, epithelial, cardiac and vascular tissues [3,9,10,11]. It is assumed that endogenous analogues of CTS serve as physiological triggers of the Na,K-ATPase signal function. Accordingly, ouabain-like [12] and marinobufagenin-like [13] CTS were identified. Endogenous ouabain, presumably synthesized in the adrenal cortex and hypothalamus, circulates normally at the sub-nanomolar concentration range. However, several conditions, such as essential hypertension, chronic salt intake, congestive heart failure, pre-eclampsia, pregnancy and affective disorders are associated with an increased level of circulating ouabain [7,14,15,16,17]. Elevation of circulating ouabain is also reported during physical exercise [18].

The Na,K-ATPase is known to play critical role in the polarity and vectorial transport across epithelial cells [19]. However, studies on cultured cells indicate its additional role in formation of tight junctions (TJ) and in regulation of TJ structure and permeability via the Na,K-ATPase-mediated modulation of claudin expression. Ouabain serves as the main pharmacological tool in these studies [20,21,22]. Claudins (27 family members) are integral proteins in the plasma membrane that form the TJ and determine paracellular permeability and barrier properties of epithelium, endothelium and mesothelium [23,24,25]. It has been shown that claudin-1, -3, -4, -5, -8 decrease the permeability of epithelium barrier. In contrast, claudin-2, -10a, -10b, -15 and -17 provide an increase in transport through paracellular pathway. The role of other claudins for tissue barriers has not been established yet [26].

The ability of nanomolar concentrations of ouabain to affect the expression of claudins and epithelium barrier properties by triggering cSrc/Erk1/2 intracellular signal pathways was demonstrated previously in cell culture studies [20,21,22]. However, nothing is known about a modulation of claudins expression and TJs barrier properties by circulating ouabain in vivo. Notably, a clear discrepancy between the properties of claudins studied in cell cultures and in corresponding tissues was found. This has been proposed as a result of oligomerization of claudins [27] and their cluster organization in TJs [28].

The main goals of this study were to demonstrate the modulation of epithelial barrier properties by circulating ouabain mediated via the expression of claudins; to show the isoform-specificity of this modulation in the tissues that differ in claudin expression, their barrier properties and functional specialization. Thus, jejunum and colon were chosen because of different function and claudin expression [29]. Cerebrovascular endothelium in the frontal lobes was selected as a highly impermeable tissue [30]. We subjected rats to 4-day injections of ouabain (1 μg/kg) alone and in a combination with lipopolysaccharide (LPS) to induce acute injury; in some experiments, rats were also subjected to disuse by hindlimb suspension (HS) for 6 h. LPS and HS interventions were used to test the potential contribution of circulating ouabain to inflammatory and disuse responses. We analyzed the transepithelial resistance (TER) and claudin expression in the jejunum and colon; claudin expression in brain frontal lobes. In porcine jejunum cell line, IPEC-J2, the effect of nanomolar ouabain concentration on TER and claudin expression as well as cSrc-kinase activation by phosphorylation were examined.

## 2. Results

### 2.1. Low Ouabain Concentration Stimulates Epithelial Barrier Formation in IPEC-J2 Cells

Previously, in cell cultures, effects of nanomolar ouabain concentrations on claudin-1, -2, -4 and -11 expression were investigated [20,21,22]. In this study, we used the porcine jejunum cell line IPEC-J2 [31] to test the effect of nanomolar ouabain on the expression of a broad range of claudins.

**Figure 1:**
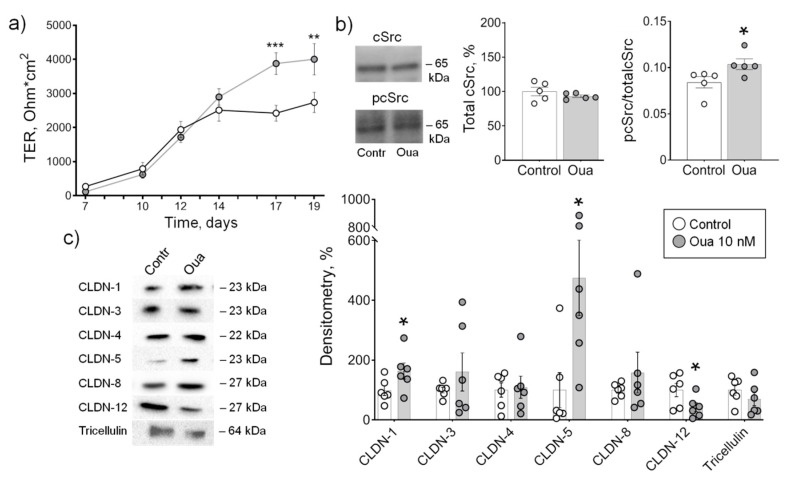
Epithelial barrier formation in IPEC-J2 cells grown in control medium and in the presence of 10 nM ouabain (Oua). After 7 days of culture, transepithelial resistance (TER) values were determined every 2–3 days using an EVOM volt-ohmmeter; after 19 days, cells were used for subsequent analysis. (**a**) Transepithelial resistance (TER) (*n* = 6 for each group). (**b**) The expression level of total cSrc (central plot, the relative expression of cSrc protein shown as a percentage of average level in control samples taken for 100%) and the cSrc-kinase activation by phosphorylation (right plot, shown as a ratio between immunoblot intensity corresponding to phosphorylated pcSrc over total cSrc, as it is shown in the representative immunoblots in left panel) (*n* = 5 for each group). (**c**) Western blot analysis of claudin (CLDN) and tricellulin expression (*n* = 6 for each group); left panel shows representative immunoblots. Original images for Western blots using Stain-Free gels as a loading control are shown in Supplemental Materials. The number of symbols corresponds to the number of samples. One-way ANOVA with Dunnett correction: * *p* < 0.05, ** *p* < 0.01 and *** *p* < 0.001 compared with the corresponding control.

In the control medium, a gradual increase in TER was observed from 7 to 19 days of IPEC-J2 cell growth. In the presence of 10 nM ouabain, there was a significant increase in TER at 17 and 19 days of incubation compared with the control (Figure 1a) that confirms our previous observations [32]. The level of cSrc-kinase activation by tyrosine auto-phosphorylation was significantly increased at 19 days of incubation with ouabain (Figure 1b). Importantly, the level of total cSrc was not affected by chronic ouabain exposure suggesting that the increased cSrc activation was a result of signaling but not expressional changes of cSrc. The increase in TER and cSrc activation was accompanied with an increase in the expression of tightening claudin-1, -5 and down-regulation of pore-forming claudin-12 (Figure 1c). The expression of tightening claudin-3, -4, -8 and tricellulin was not changed.

### 2.2. Chronic but Not Acute Ouabain Administration Modulates Intestine Epithelium Barrier Properties

**Figure 2:**
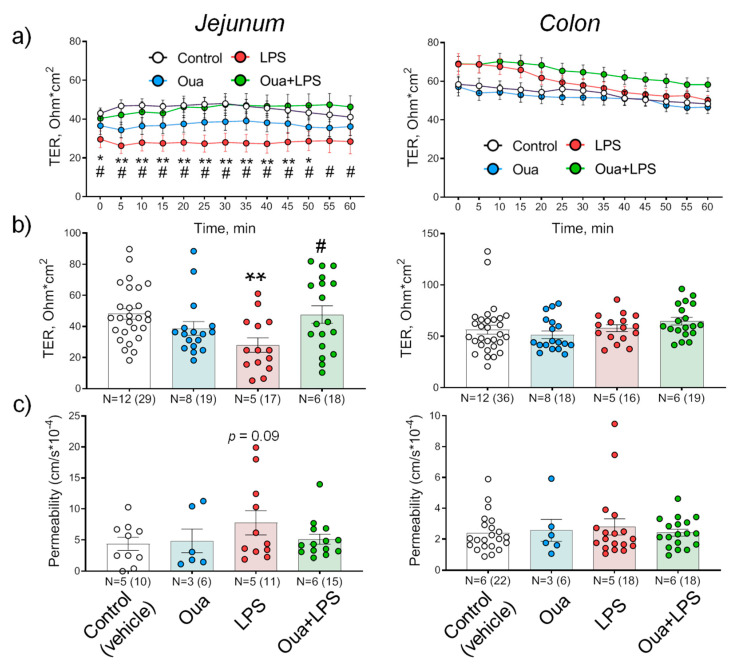
Effects of chronic ouabain (Oua) and lipopolysaccharide (LPS) administration on the barrier properties of rat jejunum and colon. Rats were intraperitoneally injected with ouabain (1 µg/kg) for 4 days. (**a**) Transepithelial resistance (TER) dynamics. (**b**) TER values measured at 30 min of registration. (**c**) Apparent permeability measured as the paracellular flux of sodium fluorescein. N—the number of rats (in parentheses is the number of fragments). The number of symbols corresponds to the number of fragments. One-way ANOVA with Dunnett correction: * *p* < 0.05 and ** *p* < 0.01—LPS-treated group compared with the corresponding control (vehicle treated group). # *p* < 0.05—Oua + LPS compared with LPS-treated group. *p* = 0.09 in (**c**) corresponds to comparison with the control.

In the control, at 30 min of registration, TER values of the jejunum and the colon were 50 ± 4 Ohm·cm^2^ (*n* = 23) and 57 ± 4 Ohm·cm^2^ (*n* = 30), respectively, and remained stable during 60 min of the experiment (Figure 2a,b). Chronic administration of ouabain (1 µg/kg) significantly (*p* < 0.05) increased the level of circulating ouabain in rat serum from 2.6 ± 0.3 nM in control up to 4.7 ± 1.5 nM (see also [33]). Ouabain treatment did not significantly affect TER of both jejunum and colon (Figure 2a,b). Accordingly, the paracellular flux of sodium fluorescein, which reflects TJ restriction to organic molecules also was not changed (Figure 2c).

**Figure 3:**
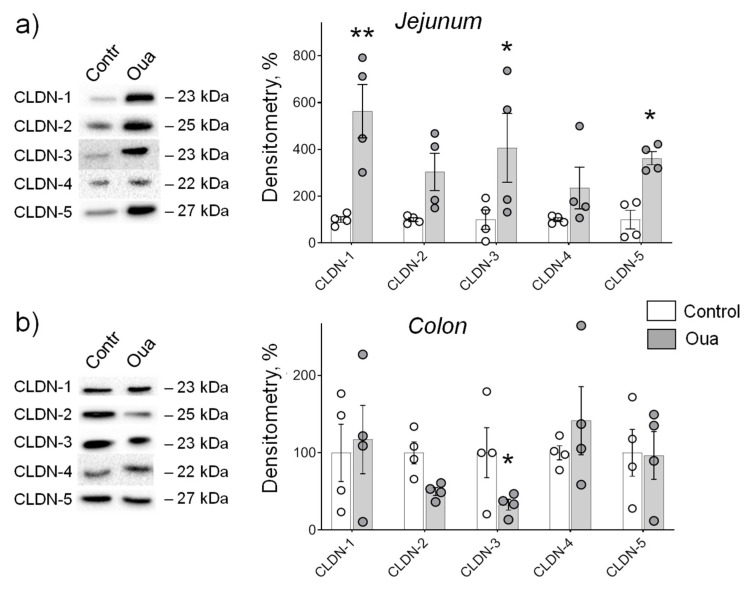
Chronic exposure to ouabain (Oua) differently alters claudin expression in rat jejunum (**a**) and colon (**b**). Rats were intraperitoneally injected with ouabain (1 µg/kg) for 4 days. Western blot analysis of claudins (CLDN) expression (*n* = 4 for each group); left panel shows representative immunoblots. Original images for Western blots using Stain-Free gels as a loading control are shown in Supplemental Materials. The number of symbols corresponds to the number of samples. One-way ANOVA with Dunnett correction: * *p* < 0.05 and ** *p* < 0.01 compared with the corresponding control (vehicle treated group).

In the jejunum, chronic ouabain exposure was accompanied with a significant increase in the expression of claudin-1, -3, -5 without changes in claudin-2, -4 (Figure 3a). In the colon, only claudin-3 was affected, however, in contrast to the jejunum, the decreased expression of claudin-3 was seen (Figure 3b). These data indicate that chronic ouabain tissue-specifically modulates claudin expression in rat intestine. This regulation is most pronounced in the jejunum and claudin-3 expression can be both up- and down-regulated in the jejunum and colon, respectively.

**Figure 4:**
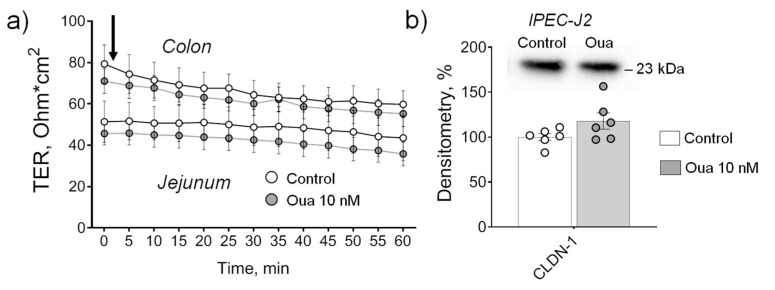
Acute application of ouabain (Oua, 10 nM) to the basolateral side does not alter transepithelial resistance (TER) of rat jejunum and colon (**a**) as well as claudin-1 expression in IPEC-J2 cells (**b**). (**a**) Vertical arrow indicates the time of ouabain addition. Each data point represents the mean of measurements from 4–8 intestine fragments obtained from 4 rats. (**b**) Western blot analysis of claudin-1 (CLDN-1) expression (*n* = 6 for each group); upper panel shows representative immunoblots. After 19 days of culture, non-treated IPEC-J2 cells were incubated in the absence (control medium) or in the presence of 10 nM ouabain added for 240 min with subsequent Western blot analysis. Original images for Western blots using Stain-Free gels as a loading control are shown in Supplemental Materials. The number of symbols corresponds to the number of samples. One-way ANOVA with Dunnett correction.

It has been reported previously that 60-min exposure to 10 nM ouabain activates both cSrc and ERK1/2 kinases, and increases gap junctional communication in Madin–Darby canine kidney (MDCK) cells [34]. In this study, ouabain (10 nM) acutely applied to the basolateral side of jejunum and colon, which were isolated from non-treated rats, did not alter TER within 60 min of incubation (Figure 4a).

Similarly, ouabain (10 nM) also did not alter TER within 240 min of incubation (data not shown) as well as claudin-1 expression in control (non-treated) monolayers of IPEC-J2 cells (19 days of cultivation, the protocol similar to the experiments in part Section 2.1) (Figure 4b).

### 2.3. Chronic Ouabain Administration Protects against Intestine Function Injury

LPS is a glycolipid present in outer membrane of Gram-negative bacteria that triggers production of inflammatory mediators. Systemic administration of LPS is widely used as a model of bacterial sepsis. LPS is known to increase intestinal epithelial TJ permeability and plays an important role in mediating intestinal inflammatory responses [35]. Recently, a protective effect of ouabain against LPS-induced acute lung injury in mice was shown [36]. 

In this study, LPS (1 mg/kg) decreased TER as well as increased sodium fluorescein transport in the jejunum confirming the increased TJ permeability and disturbance of barrier properties. Ouabain (1 µg/kg) pre-treatment for 4 days completely prevented these LPS-induced disturbances (Figure 2 a-c). In contrast, the colon was resistant against both LPS-induced injury as well as chronic ouabain pre-treatment (Figure 2a–c).

Prolonged exposure to microgravity [37] or HS (a well-accepted animal ground-based spaceflight model) [38,39] are known to impair intestinal function that reduces its resistance to bacterial infection [39]. It was recently shown that the first 6 h of HS disturbs rat postural soleus muscle function and depolarizes the muscle membrane, while pre-treatment with ouabain (1 µg/kg) protects against this disturbance [33]. The effect of an acute HS on the gut function was not assessed yet. 

**Figure 5:**
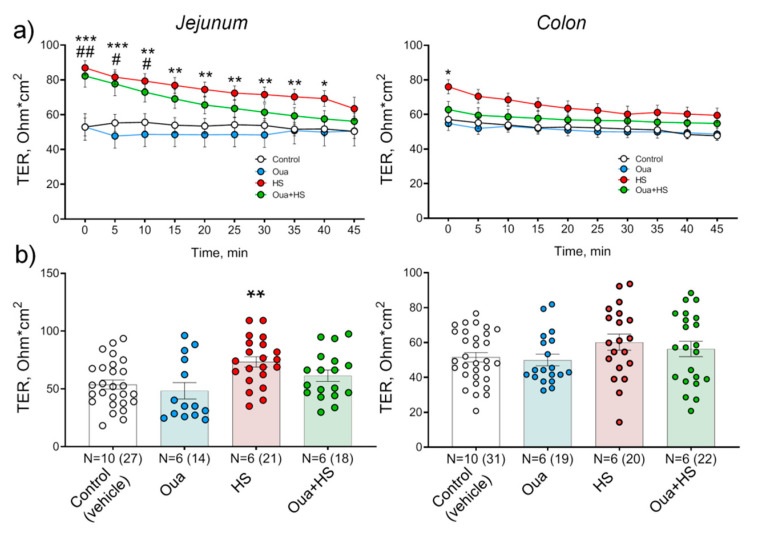
Effects of chronic ouabain (Oua) administration and hindlimb suspension (HS) on barrier properties of rat jejunum and colon. Rats were intraperitoneally injected with ouabain (1 µg/kg) for 4 days. (**a**) Transepithelial resistance (TER) dynamics. (**b**) TER values measured at 30 min of registration. N—the number of rats (in parentheses is the number of fragments). The number of symbols corresponds to the number of fragments. One-way ANOVA with Dunnett correction: * *p* < 0.05, ** *p* < 0.01 and *** *p* < 0.001—HS group; # *p* < 0.05 and ## *p* < 0.01—Oua + HS group compared with the corresponding control (vehicle treated group).

In this study, TER of the control jejunum and colon remained stable during whole experiment duration (45 min) (Figure 5a). After 6 h of HS, the initial TER of both jejunum and colon was significantly higher compared to the control suggesting disturbance of barrier properties of these tissues. Moreover, a pronounced decline of TER values was observed over time (Figure 5a). In the jejunum, ouabain (1 µg/kg) pre-treatment for 4 days accelerated TER decline to control level compared with HS conditions only (Figure 5a,b). In contrast, the colon was less sensitive to HS-induced injury and insensitive to chronic ouabain pre-treatment (Figure 5a,b).

### 2.4. Chronic Ouabain Administration Modulates Claudin Expression in Cerebral Blood Vessels

**Figure 6:**
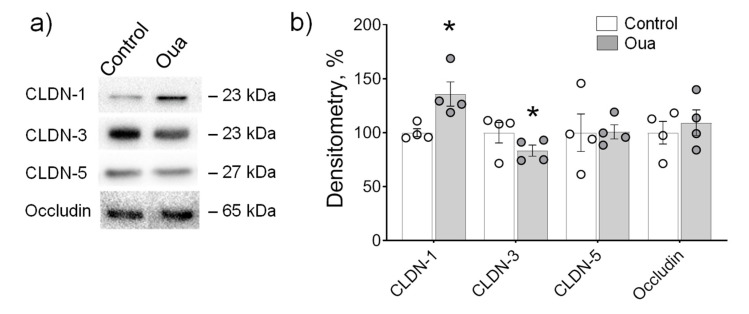
Chronic exposure to ouabain (Oua) altered claudin expression in rat brain frontal lobes. Rats were intraperitoneally injected with ouabain (1 µg/kg) for 4 days. (**a**) Representative immunoblots. (**b**) Western blot analysis of claudin (CLDN) and occludin expression (*n* = 4 for each group); Original images for Western blots using Stain-Free gels as a loading control are shown in Supplemental Materials. The number of symbols corresponds to the number of samples. One-way ANOVA with Dunnett correction: * *p* < 0.05 compared with the control (vehicle treated group).

A vascular endothelium of rat brain frontal lobes was used as a model of tissue with relatively high barrier tightness. TJ proteins, i.e., tightening claudin-1, -3, -5 and occludin that underlie the organization of the blood–brain barrier, have been identified previously in cerebrovascular endothelium [40,41,42]. In our study, all these proteins were identified. Chronic administration of ouabain (1 µg/kg) significantly increased the expression of claudin-1 and decreased claudin-3, while claudin-5 and occludin were not altered (Figure 6a,b).

## 3. Discussion

An endogenous analogue of a specific ligand of the Na,K-ATPase, ouabain, has been suggested to act as a hormone that circulates under normal conditions at sub-nanomolar concentration range. The unique role of endogenous ouabain in cardiovascular and central nervous systems, kidney and other organs in health and disease is well documented [7,14,16,17]. Claudins are the major proteins that determine paracellular permeability and barrier properties of the epithelium [28,43,44]. Previous studies on cell cultures suggest the ability of nanomolar ouabain to regulate the formation, structure and permeability of TJ via Na,K-ATPase-mediated modulation of claudin expression [20,21,22].

This study tested our hypothesis that chronic elevation of circulating ouabain in vivo can affect the expression of claudins in different tissues. Our results provide several novel findings:

(1) Claudins of rat intestine and brain blood vessels as well as IPEC-J2 cells are subjected to regulation by chronic ouabain exposure.
(2) Claudin-1 is specifically up-regulated by ouabain in contrast to other claudins that demonstrated a variety of tissue-specific changes.
(3) Chronic ouabain differently affects claudin expression in rat jejunum and colon.
(4) During LPS- or HS-induced injury, the jejunum is predominantly targeting by circulating ouabain and ouabain pre-treatment prevents the functional impairment in this tissue; the colon is relatively resistant to these injuries alone and in a combination with ouabain pre-treatment.

Epithelial cells express mostly α1 isoform of the Na,K-ATPase, except testes and spermatocytes that also co-expresses the α4 isoform [1]. Currently, a variety of observations corroborate the functional interaction between the Na,K-ATPase and claudins and its possible role in the physiological mechanism of epithelial phenotype regulation by endogenous ouabain [20,21,22]. The mechanism behind action of low ouabain concentrations in different tissues is not completely understood. It remains uncertain whether binding of ouabain affects cellular functions by inhibiting enzymatic activity and, thus, altering ion homeostasis, or by conformational changes of the α subunit that initiate signal transduction.

The Na,K-ATPase α subunit is the only known receptor for ouabain [8]. The Na,K-ATPase is now considered as an important signaling molecule that can transduce ouabain binding into an activation of downstream signaling. Binding of ouabain activates the Na,K-ATPase/cSrc complex resulting in initiation of protein kinase cascades and production of second messengers that alter cellular functions in a cell-specific manner [9,10,11]. Previous studies on MDCK II (Madin–Darby canine kidney), rat Sertoli (the key structure of blood-testis barrier) and ADPKD (autosomal dominant polycystic kidney disease) cell cultures demonstrated that nanomolar ouabain induces the expression of claudin-1, -2, -4 and -11 [20,21,22]. These studies suggest the cSrc/Erk1/2-dependent intracellular signaling that regulates epithelial phenotype and proliferation upon ouabain binding [11,20,21,22]. We used the porcine jejunum cell line IPEC-J2, which is considered as a reliable model for studying human gastrointestinal tract [31]. This IPEC-J2 cell study further indicates that claudin-1, -5 and -12 are also subjected to regulation by ouabain. Notably, both up-regulation (tightening claudin-1, -5) and down-regulation (pore-forming claudin-12) is occurring. This was accompanied with increased level of cSrc-kinase activation. Altogether, this suggests that the most likely mechanism for the expression modulation of claudins is the ouabain-induced cSrc kinase signaling pathway.

However, processes other than cSrc-dependent mechanisms can also be involved. Although ouabain is the specific inhibitor of the Na,K-ATPase, the ability of ouabain to activate the α1 Na,K-ATPase at concentrations comparable to its endogenous level was shown [45,46,47,48]. The mechanism of this stimulation is debated. The direct activation by low ouabain concentrations was suggested for endothelial α1 Na,K-ATPase [47]. Alternatively, ouabain-mediated stimulation of the α1 Na,K-ATPase in renal cells requires specific molecular environment and can be modulated by an initial increase in intracellular Na^+^ resulting from Na,K-ATPase inhibition by endogenous ouabain [45,46]. Moreover, ouabain-induced signal transduction might be mediated by pathways triggered by imbalance in [Na]_i_/[K]_i_ ratio [47,48,49]. Further studies are needed to reveal a mechanistic background of ouabain-induced modulation of claudin expression.

Among different tissue-specific claudin expression patterns, tightening claudin-1 is ubiquitously expressed [23,29,50,51]. In experiments employing confluent monolayers, nanomolar ouabain has been previously shown to increase the expression of claudin-1, -2 and -4 in MDCK II cells [20], claudin-1 and -11 in Sertoli cells [21] and claudin-1 in ADPKD cells [22]. In our study, nanomolar ouabain affected the process of epithelium barrier formation in IPEC-J2 cells accompanied with increased expression of claudin-1, -5 and down-regulation of claudin-12. In the rat jejunum, chronic elevation of circulating ouabain increased the expression of claudin-1, -3 and -5; in the cerebral blood vessels, claudin-1 up-regulation was associated with the reduction in claudin-3.

Thus, to our knowledge, all studies including our observations suggest that low ouabain concentrations specifically up-regulate claudin-1, while other claudins respond in a tissue-specific manner. These intriguing data suggest an unique of Na,K-ATPase/claudin-1 functional interaction that is not seen for other claudins. It was previously shown that nanomolar ouabain modulates the TJ of MDCK cells in a very complex manner, where claudin-1 and -4 are controlled via different signaling pathways [20]. The nature of this phenomenon is unknown. Currently, we can only speculate that such interaction requires a specialized subcellular localization and interactions of these proteins with the molecular environment. The ability of claudins for oligomerization [27] and cluster organization [28] should also be considered. Particularly, cholesterol is a key molecule in the formation of specialized membrane microdomains i.e., lipid rafts, which are known as a molecular platform involved in numerous cellular processes. Membrane cholesterol was shown to stabilize the association of TJ proteins in epithelial monolayers in Caco-2 cells. Notably, depletion of membrane cholesterol resulted in the displacement of claudin-3, -4, -7 and occludin from the cholesterol rich domains associated with TJs, while claudin-1 was unaffected [52]. Intriguingly, a neuroprotective effect of ouabain against LPS-induced oxidative stress in rat cerebellum involves promoting membrane lipid remodeling [53]. Further studies are necessary to testify features of the Na,K-ATPase/claudin-1 functional interaction and to identify their precise molecular basis.

Our observations suggest that chronic ouabain administration protects against intestine function injury. Ouabain pre-treatment for 4 days completely prevented LPS-induced jejunum TER disturbances (Figure 2). Studies of regulatory and signaling processes in the early (within hours) stages of adaptation to microgravity are mainly carried out in relation to postural muscles [54]. Our findings suggest that gut functional disturbances also belong to the earliest remodeling events induced by simulated microgravity. Epithelium TJs are known as a sensor of osmolality and hydrostatic pressure gradients [55,56]. Thus, HS-induced TER abnormalities (Figure 5) can result from changes in the hydrostatic pressure gradient in the abdominal cavity of hindlimb suspended rats, when radical redistribution of body fluids occurs with possible changes in the water–salt balance. Balanced hydrostatic pressure on both sides of the tissue in the Ussing chamber eliminates this factor and provides restoration of epithelium barrier functions.

Taken together, our data suggest that intestine barrier properties can be regulated by circulating ouabain and this regulation is pronounced under functional impairments such as LPS- and HS-induced injury of intestine function. Notably, this regulation is characteristic for the jejunum only.

Previous observations suggest that mouse colon is relatively resistant to such injury as radiation compared to the jejunum [57]. Other data also demonstrated that cholera toxin differently affected claudin expression in rat colon [51] and jejunum [58]. In our study, the expression of claudins in rat colon was relatively resistant to chronic ouabain exposure as well as to LPS- or HS-induced injury in a combination with ouabain pre-treatment (TER measurements). In contrast, jejunum was significantly affected by these interventions. The reason for different efficacy of chronic ouabain in the jejunum and colon is unknown. One can only assume that functional differences between these parts of intestine might be the reason. This includes the differences in expressed claudins [29].

Potential role of very low ouabain concentrations in neural tissues is well documented. Several previous reports suggest that circulating ouabain modulates neuronal functions and may be involved in depressive disorders [14,59]. Neutralization of endogenous ouabain using anti-ouabain antibodies administration elicits anti-depressive behavior suggesting that alteration in circulating ouabain may be of significant therapeutic value [59]. Anti-apoptotic effects were described when low ouabain doses were injected in vivo into the rat brain [60]. Low-doses of ouabain also protected hippocampal slice cultures from experimental ischemia [61]. It was shown in primary culture of rat cortical neurons that ouabain at sub-nanomolar concentrations can prevent Ca^2+^ overload and neuronal apoptosis in excitotoxic stress [62]. The same doses of chronically administrated ouabain used in this study (1 µg/kg–1.8 µg/kg) significantly improves mouse recovery following traumatic brain injury [63] and attenuates the oxidative stress induced by LPS in rat cerebellum [53]. The role of ouabain in inflammatory processes in the central nervous system is reviewed elsewhere [64].

In our study, chronic exposure to ouabain (1 µg/kg) increased claudin-1 expression and decreased claudin-3 in blood vessels of rat brain frontal lobes. In contrast to intestine epithelium, endothelium of cerebral blood vessels demonstrated a high degree of impermeability [30]. Thus, our data suggest that circulating ouabain targets claudins mosaic in blood–brain barrier structures.

## 4. Materials and Methods

### 4.1. Animals

Experiments were performed on male Wistar rats (180–230 g). Animals were housed in a temperature- and humidity-controlled room with food and water ad libitum. All procedures involving rats were performed in accordance with the recommendations of the Guide for the Care and Use of Laboratory Animals [65]. The experimental protocol met the requirements of the EU Directive 2010/63/EU for animal experiments and was approved by the Ethics Committee of St. Petersburg State University (issued 13 December 2017) and the Animal Experiments Inspectorate of the Danish Ministry of Environment and Food (issued 5 July 2016).

Rats were intraperitoneally injected with vehicle (0.9% NaCl) or 1 µg/kg body weight ouabain daily for 4 days as described previously [45]. In some experiments, two hours after last injection of ouabain (1 µg/kg), lipopolysaccharide (LPS, 1 mg/kg) was intraperitoneally administrated to induce acute injury. The serum level of ouabain was estimated using ELISA Kit for Ouabain (Cloud-Clone corp., Katy, TX, USA). Twenty-four hours after last injection of ouabain, jejunum, colon and brain frontal lobes were isolated. In separate experiments, twenty-four hours after the last ouabain injection, rats were subjected to HS, widely used as an animal model of disuse that leads to progressive atrophy of postural skeletal muscles. The rats were subjected to HS individually in custom cages for 6 h, as described previously [66]. Control animals were not suspended. In these experiments, jejunum and colon were isolated. Freshly isolated jejunum and colon were immediately used for electrophysiological experiments. For later biochemical assays, some tissues were snap-frozen in liquid nitrogen and then stored at −80 °C.

In a separate set of experiments, ouabain was acutely added to freshly isolated jejunum and colon obtained from non-treated rats.

### 4.2. Transepithelial Electrical Resistance Recording

Ussing chamber technique was used to measure TER as reported previously [67]. Briefly, colon and jejunum fragments were mounted in Ussing chambers (exposed area: 0.13 cm^2^), and bathed with solution containing (in mM) NaCl, 119; KCl, 5; CaCl_2_, 1.2; MgCl_2_, 1.2; NaHCO_3_, 25; Na_2_HPO_4_, 1.6; NaH_2_PO_4_, 0.4; d-glucose, 10; (pH 7.4). The solution was gassed with 95% O_2_ and 5% CO_2_ and heated to 37 °C. To evaluate TER, we recorded voltage changes to 10 μA current and calculated it using Ohm’s law.

### 4.3. Epithelium Permeability Measurements

Sodium fluorescein was used to study permeability of epithelial paracellular pathway for macromolecules, as reported previously [68]. Sodium fluorescein has a molecular mass of 376 Da, is electrically neutral and diffuses through the epithelium along the paracellular path with concentration gradient. Once the tissue was mounted in the Ussing chamber, 50 μL of the apical solution was replaced with 50 μL of similar solution containing sodium fluorescein. The final concentration of sodium fluorescein in the chamber was 100 μM. Solutions from the basolateral side were analyzed after 60 min of incubation. The concentration of sodium fluorescein in the samples was determined using Typhoon FLA 9500 high-resolution laser scanner (GE, Piscataway, NJ, USA). The wavelengths of laser excitation and absorption were 473 and 510 nm, respectively. Analysis of the obtained data was performed using the ImageJ program (NIH, Rockville Pike, Bethesda, MD, USA).

### 4.4. Cell Culture

IPEC-J2 cell culture was obtained from DSMZ-German Collection of Microorganisms and Cell Cultures (Braunschweig, Germany). IPEC-J2 cells were seeded and cultured in DMEM/F12 medium (1:1) with stable glutamine (Biochrom, Berlin, Germany) supplemented with 10% porcine serum and 1% Penicillin-Streptomycin (Biochrom, Berlin, Germany) at 37 °C in a humidified atmosphere of 5% CO_2_. For incubation, the cells were detached with Trypsin/EDTA (0.05%, 0.02%) (Sigma-Aldrich, Taufkirchen, Germany). After centrifugation at 200 g for 5 min, the supernatant was discarded and the pellet was resuspended in cell culture medium with or without 10 nM of ouabain (and diluted to a final concentration of 2 × 10^5^ cells/mL). Cells were seeded on Millicell-PCF cell culture inserts (Merck Millipore Ltd., Darmstadt, Germany). Medium with or without 10 nM of ouabain was changed every 2–3 days. After 7 days of culture, TER values were determined every 2–3 days using an EVOM volt-ohmmeter with a chopstick electrode (WPI, Sarasota, FL, USA). All TER values were normalized by the insert area and obtained after background subtraction. After 19 days of culture, IPEC-J2 cells were homogenized in lysis buffer containing (in mM): HEPES, 25; EDTA, 2; NaF, 25; 1% SDS, and protease inhibitors cOmplete mini EDTA-free tablets (Roche, Mannheim, City, Germany) (pH 7.6). Protein content was quantified employing Bio-Rad DC Protein Assay reagent (Bio-Rad, Hercules, CA, USA) and a plate reader (PerkinElmer, Waltham, MA, USA). Protein samples were frozen at −80 °C for subsequent determination of cSrc-kinase phosphorylation (see Section 4.5) and for Western blot analysis (see Section 4.6).

After 19 days of culture, some inserts with non-treated IPEC-J2 cells were mounted in Ussing chamber to measure TER values in the absence (control medium) or in the presence of 10 nM ouabain added for 240 min to basolateral side of the cell layer. Then, as described above, cells were homogenized in lysis buffer, protein content was quantified and samples were frozen at −80 °C for subsequent Western blot analysis.

### 4.5. cSrc-kinase Phosphorylation Measurement

Ten micrograms of total protein diluted in Laemmli sample buffer (Bio-Rad, Hercules, CA, USA) was loaded on 4–20% precast polyacrylamide Stain-Free gels (CriterionTM TGX Stain-freeTM precast gel, Bio-Rad, Hercules, CA, USA). Total protein load was detected on the Stain-Free gels using UV-light in imaging system (c600, Azur Biosystems, Dublin, CA, USA). The proteins were electrotransferred onto membranes that were then blocked by incubation in 5% bovine serum albumin and 5% nonfat dry milk in phosphate buffered saline (PBS) with 0.5% *v*/*v* Tween 20 (PBS-T). The membranes were incubated overnight at 4 °C with either antibody against total cSrc (sc-8056; Santa Cruz Biotechnology Inc., Dallas, Texas, USA) or antibody against cSrc phosphorylated at pY418 (1:200; #44660G; ThermoFisher Scientific, Waltham, MA, USA). After intensive washing, the membranes were incubated with horseradish-peroxidase (HRP)-conjugated secondary antibody (1:4000; Dako, Denmark) for 1 h in PBS-T. Excess antibody was removed by washing, and bound antibody was detected by an enhanced chemiluminescence kit (ECL, Amersham, UK). Detected total cSrc protein was normalized using the ImageJ program (NIH, Rockville Pike, Bethesda, MD, USA) as a ratio to total protein load measured in membrane for the same probe. The activation of cSrc by tyrosine phosphorylation at Y418 was quantified as a ratio of phosphorylated cSrc (pcSrc) normalized for protein load over total cSrc normalized to the corresponding protein load. 

### 4.6. Western Blot Assays

SDS buffer (Laemmli) was added to the IPEC-J2 protein extract, samples were loaded on 10% Stain-Free gels and electrophoresis was performed. Proteins were stained with primary antibodies raised against claudin-1, -3, -4, -5, -8, -12 and tricellulin and visualized using secondary goat anti-rabbit and anti-mouse IgG antibodies and chemiluminescence reaction (Bio-Rad, Hercules, CA, USA). In experiments with acute ouabain (10 nM) application, only claudin-1 expression was detected.

Frozen intestine or brain samples were homogenized in a Tris-buffer containing (in mM) Tris, 10; NaCl, 150; Triton X-100, 0.5; SDS, 0.1, protease inhibitors cOmplete mini EDTA-free tablets (Roche, Mannheim, Germany) (pH 7.6). After homogenization, samples were centrifuged and the supernatant was then cooled on ice for 30 min and retrieved after a second centrifugation step for 15 min at 15,000 g at 4 °C (Sigma-Aldrich, Munich, Germany). Protein content was quantified using Bio-Rad DC Protein Assay reagent (Bio-Rad, Hercules, CA, USA) and a plate reader (PerkinElmer, Waltham, MA, USA). SDS buffer (Laemmli) was added to extracted proteins, and samples were loaded on 10% Stain-Free gels and electrophoresis was performed. Proteins were stained with primary antibodies raised against claudin-1, -2, -3, -4, -5 for small and large intestine or claudin-1, -3, -5 and occludin for frontal lobes and visualized using secondary goat anti-rabbit IgG antibodies and chemiluminescence reaction (Bio-Rad, Hercules, CA, USA). Band intensities were measured using luminescence imager Chemi-Doc MP Imaging System (Bio-Rad, Hercules, CA, USA).

The following antibodies were used: claudin-1 (#ab56417, 1:50, Abcam, Cambridge, UK), claudin-2 (#32-5600, 1:100, Invitrogen, Carlsbad, CA, USA), claudin-3 (#34-1700, 1:100, Invitrogen, Carlsbad, CA, USA), claudin-4 (#ab53156, 1:500, Abcam, Cambridge, UK), claudin-5 (#34-1600, 1:100, Invitrogen, Carlsbad, CA, USA), claudin-8 (#40-0700Z, 1:100, Invitrogen, Carlsbad, CA, USA), claudin-12 (#38-8200, 1:50, Invitrogen, Carlsbad, CA, USA), occludin (#33-1500, 1:100, Invitrogen, Carlsbad, CA, USA), tricellulin (#700191, 1:100, Life Technologies, Carlsbad, CA, USA), anti-rabbit IgG (#7074, 1:1000, Cell Signaling Technologies, Leiden, The Netherlands), anti-mouse IgG (#7076, 1:1000, Cell Signaling Technologies, Leiden, The Netherlands).

Detected proteins were normalized using the Image Lab 6.1 Software (Bio-Rad, Hercules, CA, USA) as a ratio of total protein load measured in membrane of the same sample.

### 4.7. Materials

Ouabain, LPS and other chemicals were purchased from Sigma-Aldrich.

### 4.8. Statistics

All data are given as the mean ± SEM. Statistical significance of the difference between means was evaluated using one-way ANOVA. Statistical analysis was performed using GraphPad Prism 8 software (GraphPad; San Diego, CA, USA). A probability value of *p* < 0.05 was considered statistically significant.

## 5. Conclusions

Circulating ouabain tissue- and isoform-specifically regulate both intestine and blood-brain barrier claudins suggesting the therapeutic value of this signaling.

## Supplementary Materials

The following are available online at https://www.mdpi.com/1422-0067/21/14/5067/s1.

## Abbreviations

ADPKD	autosomal dominant polycystic kidney disease
CLDN	claudin
CTS	cardiotonic steroids
HS	hindlimb suspension
LPS	lipopolysaccharide
MDCK	Madin–Darby canine kidney
OUA	ouabain
TER	transepithelial resistance
TJ	tight junction
