# Supplementary material for: Circulating Ouabain Modulates Expression of Claudins in Rat Intestine and Cerebral Blood Vessels
Source: Int J Mol Sci. 2020 Jul 17;21(14):5067. doi: 10.3390/ijms21145067 (PMC7404321; doi:10.3390/ijms21145067)
Supplement: Supplementary file 1 [file ijms-21-05067-s001.pdf]

## Supplementary data

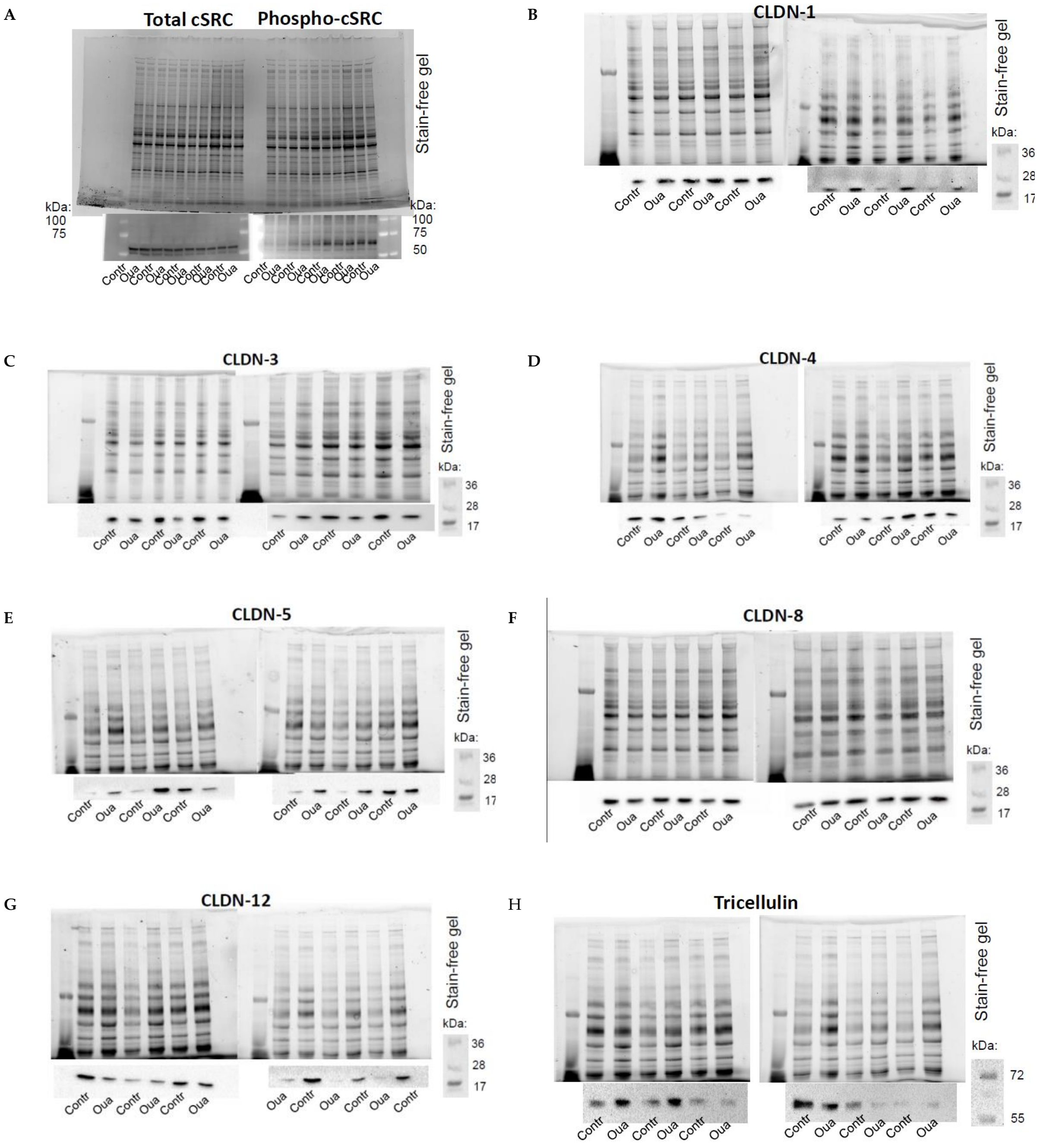


**Figure S1.** Determination of cSrc-kinase phosphorylation (**A**) and Western blot analysis of claudins (CLDN) and tricellulin (**B-H**, as indicated) expression in IPEC-J2 cells grown in control medium or in the presence of 10 nM ouabain (Oua). After 19 days of culture, IPEC-J2 cells were homogenized in lysis buffer, protein content was quantified and samples were frozen at –80 °C for subsequent analysis using Stain-Free gels as a loading control.

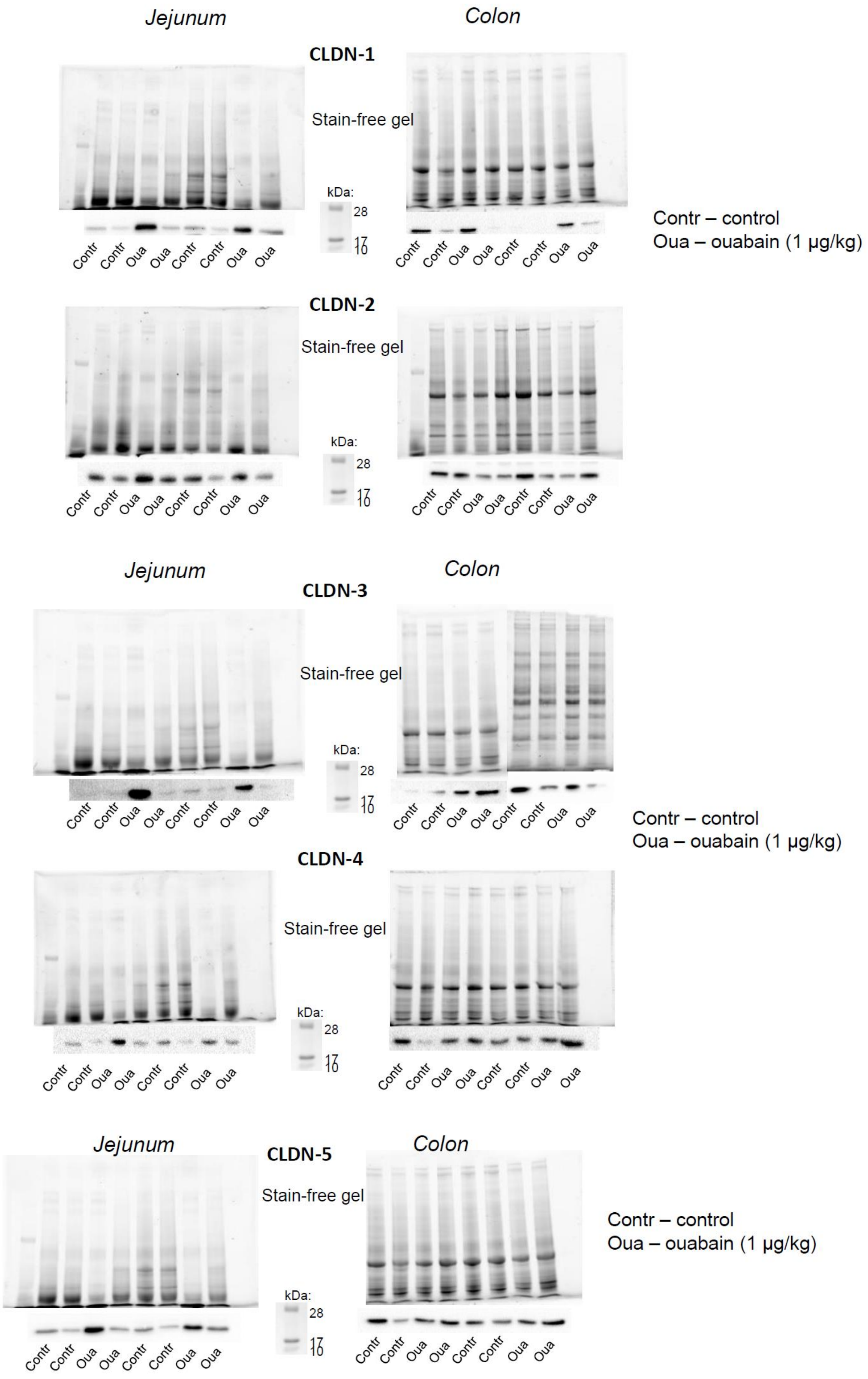


**Figure S2.** Western blot analysis of claudins (CLDN, as indicated) expression in rat jejunum and colon. Rats were intraperitoneally injected with vehicle (0.9% NaCl, control) or 1 µg/kg body weight ouabain (Oua) daily for 4 days. Twenty-four hours after last injection, isolated jejunum and colon were snap-frozen in liquid nitrogen and then stored at –80 °C for subsequent analysis using Stain-Free gels as a loading control.

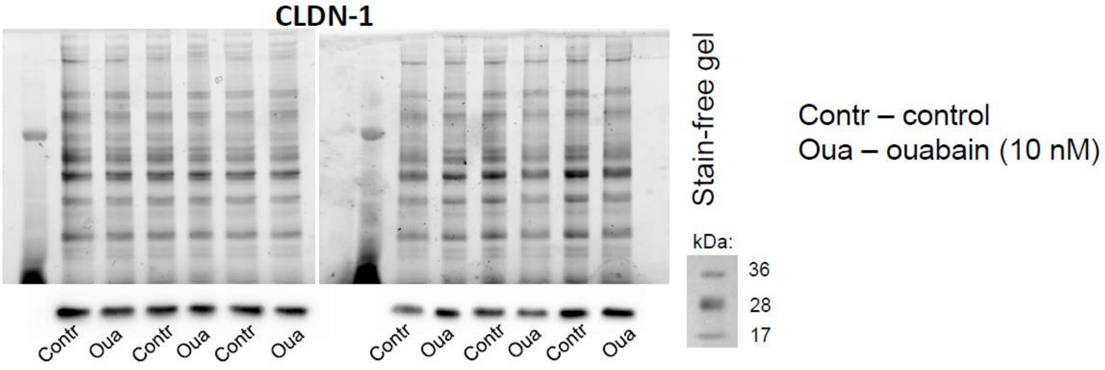


**Figure S3.** Western blot analysis of claudin-1 (CLDN-1) expression in IPEC-J2 cells grown in control medium. After 19 days of culture, IPEC-J2 cells were incubated in the absence (control medium) or in the presence of 10 nM ouabain (Oua) added for 240 min to basolateral side of the cell layer. Then, cells were homogenized in lysis buffer, protein content was quantified and samples were frozen at –80 °C for subsequent Western blot analysis using Stain-Free gels as a loading control.

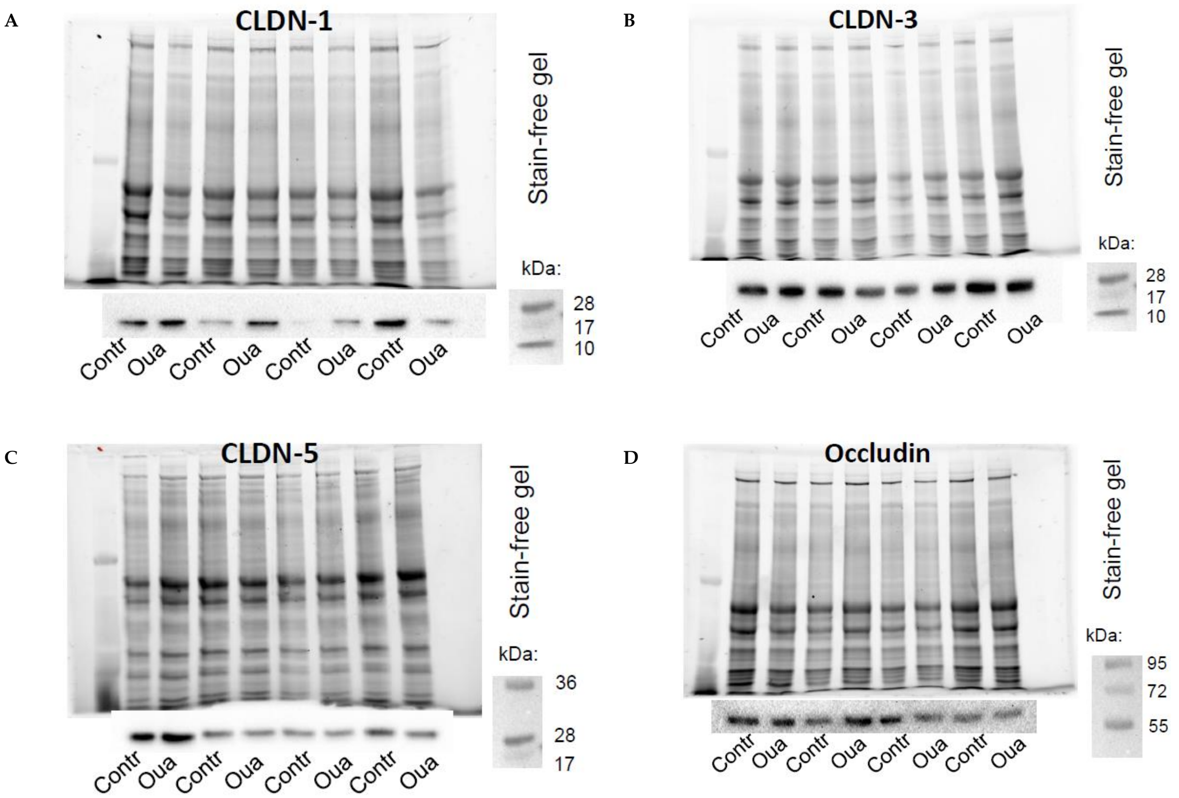


**Figure S4.** Western blot analysis of claudins (CLDN) and occludin (**A-D**, as indicated) expression in rat brain frontal lobes. Rats were intraperitoneally injected with vehicle (0.9% NaCl, control) or 1 µg/kg body weight ouabain (Oua) daily for 4 days. Twenty-four hours after last injection, isolated brain tissues were snap-frozen in liquid nitrogen and then stored at –80 °C for subsequent analysis using Stain-Free gels as a loading control.
